# Comparison of single versus fractionated dose of stereotactic radiotherapy for salvaging local failures of nasopharyngeal carcinoma: a matched-cohort analysis

**DOI:** 10.1186/1758-3284-1-13

**Published:** 2009-05-23

**Authors:** Daniel TT Chua, Shao-Xiong Wu, Victor Lee, Janice Tsang

**Affiliations:** 1 Departments of Clinical Oncology, The University of Hong Kong, Queen Mary Hospital, Hong Kong SAR, PR China; 2 Department of Radiation Oncology, Cancer Centre, Sun Yat-Sen University, Guangzhou, PR China

## Abstract

**Background:**

wLocal failure is an important cause of morbidity and mortality in nasopharyngeal carcinoma (NPC). Although surgery or brachytherapy may be feasible in selected cases, most patients with local failure require external beam re-irradiation. Stereotactic radiation using single or multiple fractions have been employed in re-irradiation of NPC, but the optimal fractionation scheme and dose are not clear.

**Methods:**

Records of 125 NPC patients who received salvage stereotactic radiation were reviewed. A matched-pair design was used to select patients with similar prognostic factors who received stereotactic re-irradiation using single fraction (SRS) or multiple fractions (SRM). Eighty-six patients were selected with equal number in SRS and SRM groups. All patients were individually matched for failure type (persistent or recurrent), rT stage (rT1-2 or rT3-4), and tumor volume (≤ 5 cc, >5–10 cc, or >10 cc). Median dose was 12.5 Gy in single fraction by SRS, and 34 Gy in 2–6 fractions by SRM.

**Results:**

Local control rate was better in SRM group although overall survival rates were similar. One- and 3-year local failure-free rates were 70% and 51% in SRS group compared with 91% and 83% in SRM group (p = 0.003). One- and 3-year overall survival rates were 98% and 66% in SRS group compared with 78% and 61% in SRM group (p = 0.31). The differences in local control were mainly observed in recurrent or rT2-4 disease. Incidence of severe late complications was 33% in SRS group vs. 21% in SRM group, including brain necrosis (16% vs. 12%) and hemorrhage (5% vs. 2%).

**Conclusion:**

Our study showed that SRM was superior to SRS in salvaging local failures of NPC, especially in the treatment of recurrent and rT2-4 disease. In patient with local failure of NPC suitable for stereotactic re-iradiation, use of fractionated treatment is preferred.

## Background

Local recurrence is an important cause of treatment failure in nasopharyngeal carcinoma (NPC). Recent advances in radiotherapy planning and delivery and the use of concurrent chemo-radiotherapy have significantly reduced the incidence of local failure in NPC, and most modern series reported an overall 5-year local control rate of 76–91% [1-5]. In patients with advanced T stage and/or bulky tumor, local failure however remains an important cause of morbidity and mortality. Although surgical resection or brachytherapy can be used as salvage treatment in selected cases of local failure, most patients require external re-irradiation for retreatment of NPC. Conventional two-dimensional radiotherapy planning and delivery was commonly used in the past for external reirradiation of NPC, but treatment outcome was generally poor with a high incidence of severe late complications [6-8]. Three-dimensional conformal radiotherapy can achieve better target coverage and sparing of critical structures, but the incidence of late complication still appears to be high after reirradiation of NPC even with the use of conformal radiotherapy [9]. The technique of stereotactic localization of target and treatment delivery has also been employed in salvaging local failures of NPC, which includes the use of single fraction of stereotactic re-irradiation (SRS) or multiple fractions of stereotactic re-irradiation (SRM). These two techniques were employed at Queen Mary Hospital in Hong Kong and Sun Yat Sen University Cancer Center in Guangzhou for re-irradiation of NPC, with adoption of SRS in the former center and SRM in the latter one. Different techniques were adopted at the two centers due to institutional preference and logistic reasons such as available machine time. Since there were no prospective studies comparing stereotactic re-irradiation using SRS or SRM, we conducted a retrospective study to compare the outcome of patients treated by SRS and SRT using a matched-pair design.

## Methods

### Selection of matched pair

This was a retrospective study comparing the outcome of patients with locally recurrent NPC treated by SRS and SRM. Records of patients who received SRS or SRM as salvage treatment of NPC at Queen Mary Hospital in Hong Kong and Sun Yat-Sen University in Guangzhou were reviewed for inclusion into the study. A matched pair study was used to select and analyze patients with similar prognostic factors from the two treatment groups. Only those patients who satisfied the following criteria were included in the matching process: history of poorly differentiated or undifferentiated carcinoma of the nasopharynx, completed a course of radical radiotherapy with or without chemotherapy, and histological proven local failure or progression of local disease documented by serial imaging. Patients who received SRS or SRM as a planned boost after external radiotherapy and those with disease elsewhere were excluded.

Individual patients from the two treatment groups were matched for important prognostic factors identified from previous studies: type of local failure (persistent disease, defined as local failure that occurred within 6 months of completion of primary radiotherapy, vs. recurrent disease, defined as local failure that occurred beyond 6 months of completion of primary radiotherapy), retreatment T stage (rT1-2 vs. rT3-4), and tumor volume (≤ 5 cc vs. > 5 – 10 cc vs. > 10 cc). Each patient in the SRS group was matched with another patient in the SRM group with respect to these factors, and only patients that were matched for all 3 factors were included in the study.

### Patient characteristics

Forty-eight patients received SRS at Queen Mary Hospital from January 1994 to June 2005 and 90 patients received SRM at Sun Yat-Sen University Cancer 2005 and 90 patients received SRM at Sun Yat-Sen University Cancer Center from September 1999 to December 2005 for isolated local failures of NPC. Thirteen patients were not included in the matching process due to presence of synchronous nodal disease in 2 and distant metastases in 3, and the use of SRS/SRM as planned boost in 8. The remaining 125 patients were included in the matching process, and 43 matched pairs were selected for comparison. All these patients had undifferentiated type of nasopharyngeal carcinoma and were staged by computed tomography and magnetic resonance imaging at the time of diagnosis. First course of radiotherapy was delivered using megavoltage radiotherapy with conventional two-dimensional technique, and the dose to nasopharynx was 66–70 Gy. About 23% of patients from Queen Mary Hospital and 26% from Sun Yat-Sen University Cancer Center also received adjunctive chemotherapy. All patients were jointly assessed by radiation oncologist and head and neck surgeon for other options including nasopharyngectomy and brachytherapy prior to stereotactic radiotherapy. Table 1 summarizes the patient characteristics of matched SRS and SRM groups.

**Table 1:** Characteristics of patients treated by stereotactic reirradiation using single and multiple fractions and for local failures of nasopharyngeal carcinoma

	Stereotactic radiotherapy with single fraction (n = 43)	Stereotactic radiotherapy with multiple fractions (n = 43)	All (n = 86)
Gender			
Male	32 (74%)	35 (81%)	67 (78%)
Female	11 (26%)	8 (19%)	19 (22%)

Age			
≤ 45	21 (49%)	21 (49%)	42 (49%)
> 45	22 (51%)	22 (51%)	44 (51%)
median (range)/years	46 (32–84)	46 (18–69)	46 (18–84)

Type of failure			
Persistent disease	19 (44%)	19 (44%)	38 (44%)
Recurrent disease	24 (56%)	24 (56%)	48 (56%)

Retreatment T stage			
rT1	25 (58%)	23 (54%)	48 (56%)
rT2	5 (12%)	7 (16%)	12 (14%)
rT3	9 (21%)	6 (14%)	15 (17%)
rT4	4 (9%)	7 (16%)	11 (13%)

Time from 1^st ^course of radiotherapy to reirradiation			
≤ 12 months	23 (53%)	24 (56%)	47 (55%)
> 12 – 24 monhts	3 (7%)	8 (18%)	11 (13%)
> 24 – 48 months	6 (14%)	6 (14%)	12 (14%)
> 48 months	11 (26%)	5 (12%)	16 (18%)
median (range)/months	10 (3 – 197)	10 (3 – 107)	10 (3 – 197)

Tumor volume			
≤ 5 cc	21 (49%)	21 (49%)	42 (49%)
> 5 – 10 cc	13 (30%)	13 (30%)	26 (30%)
> 10 cc	9 (21%)	9 (21%)	18 (21%)
median (range)/cc	5.1 (1.3 – 30.7)	5.6 (0.8 – 24.7)	5.2 (0.8 – 30.7)

### SRS and SRM treatment

**Figure 1:**
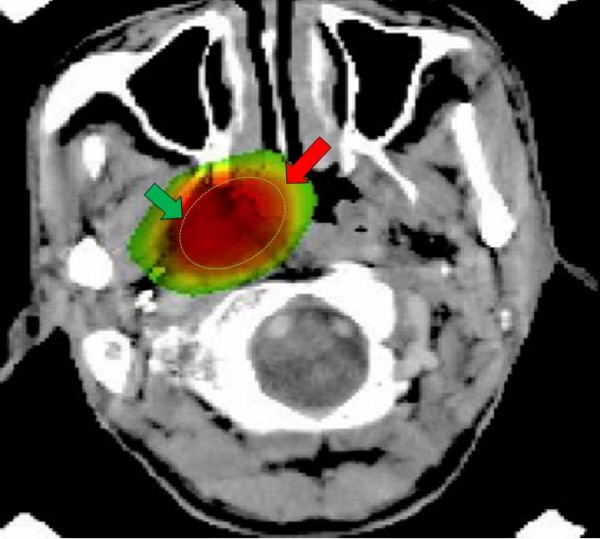
**Isodose curve showing coverage of the tumor in right side of nasopharynx in a patient treated by single fraction of stereotactic radiotherapy (target: green arrow; 80% isodose line: red arrow)**.

SRS was performed at Queen Mary Hospital using the commercial XKnife system (Radionics, Burlington, MA) to deliver multiple non-coplanar arcs of photon to the target with a modified 6 MV linear accelerator (Varian Clinac 600C, Milpitas, CA.). Head immobilization and target localization were performed with the Brown-Roberts-Wells head frame and stereotaxic system (Radionics, Burlington, MA). Axial contrast CT with a slice thickness of 2.5 to 3 mm was performed for treatment planning, supplemented by axial contrast MR with a slice thickness of 3 mm in rT3-4 disease. Target volume was defined as any abnormal soft-tissue mass and/or contrast-enhancing areas as shown in axial imaging plus a margin of about 2–3 mm. In most patients (43%), the target was covered by single isocenter using 3 to 5 arcs of beams with a degree of 90 to 210. Median dose was 12.5 Gy prescribed to 80% isodose line, with a range of 8 to 18 Gy. Figure 1 shows isodose coverage of tumor in a patient treated by SRS.

**Figure 2:**
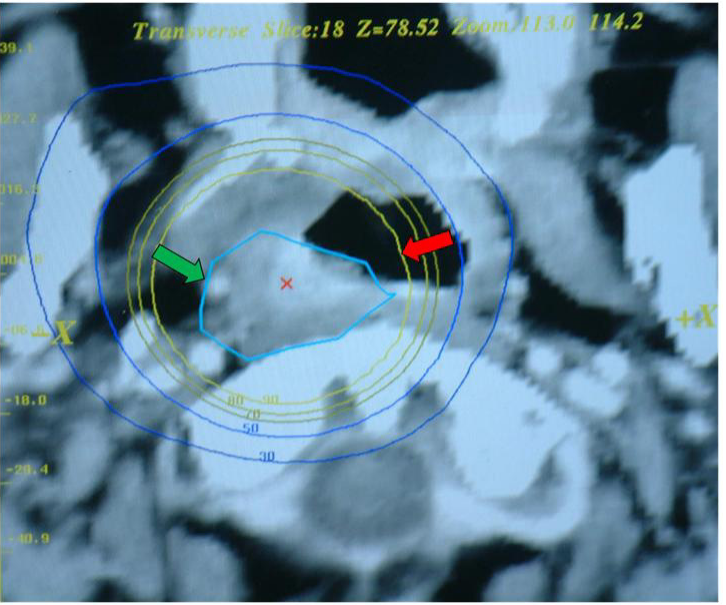
**Isodose curve showing coverage of the tumor in right side of nasopharynx in a patient treated by multiple fractions of stereotactic radiotherapy (target: green arrow; 90% isodose line: red arrow)**.

SRM was performed at Sun Yat-Sen University Cancer using a commercial stereotactic radiotherapy system (Creat, China) to deliver multiple arcs of photon with a modified 8-MV linear accelerator (Elekta, Sweden). All patients were immobilized using a relocatable head ring and bracket with a plastic mask to cover whole head. Axial contrast-enhanced computed tomography (CT) scan with a slice thickness of 3 mm was performed for treatment planning. Majority of patients only had CT performed for localization of target/critical structures and planning since MRI was not available in the center in Guangzhou before 2003. Target volume was defined by abnormal contrast-enhanced mass plus a margin of about 2 – 3 mm. The target volume was usually covered by one isocenter (98%) using four to six arcs with a degree of 30 – 150. SRT was carried out using single fraction per day and 2 – 3 fractions per week, with an inter-fractional interval of at least 1 day. Median dose was 18 Gy prescribed to 90% isodose line (range: 10 – 24 Gy) in 2 to 4 fractions for persistent disease, and 48 Gy to 90% isodose line (range: 20 – 49 Gy) in 4 – 6 fractions for recurrent disease. Figure 2 shows the isodose coverage of tumor in a patient treated by SRM.

### Response assessment and follow-up

Nasopharyngoscopy +/- biopsy and imaging were performed at 8–12 weeks after treatment to document local disease status. Patients with controlled local disease were regularly followed up every 2–3 months in the first year and every 3–4 months thereafter. Computed tomography and/or magnetic resonance imaging were performed at least annually for 3 years after treatment.

### Statistical analysis

Categorical variables were compared using chi square test or Fisher's exact test as appropriate, and continuous variables were compared using Student's t test. Treatment outcome of SRS and SRM groups were compared using the following endpoints: local failure-free rate, nodal failure-free rate, distant failure-free rate, failure-free rate and overall survival rate. The endpoints were analyzed using the product-limit method of Kaplan and Meier, and time was measured from the date of SRS or SRM until time of event occurrence, or most recent follow-up for censored observations. In patients with complete regression of disease after SRS or SRT, local failure was defined based on positive biopsy and/or radiological evidence of relapse. In patients who failed to achieve complete regression of disease after salvage treatment, local failure-free interval was set to zero. Likewise, neck node recurrence was used to define nodal failure-free rate, distant metastases was used to define distant failure-free rate, and any failure (loco-regional or distant) was used to define failure-free rate. In determining overall survival rate, event was defined as deaths due to any cause. Actuarial curves were compared between SRS and SRM groups and the significance of differences was calculated using log rank test, a p value less than 0.05 was considered to be statistically significant.

## Results

### Tumor control and survival

**Figure 3:**
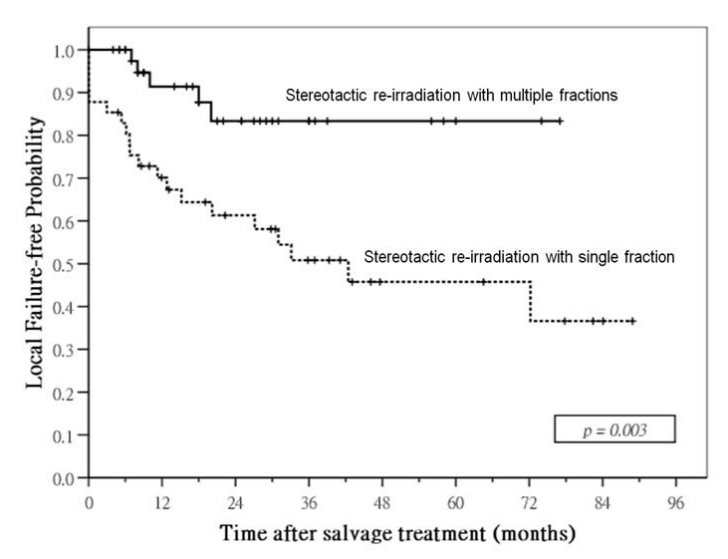
**Local control curves**. comparison of local failure-free probabilities in patients with local failures of nasopharyngeal carcinoma treated by stereotactic radiotherapy using single or multiple fractions

**Figure 4:**
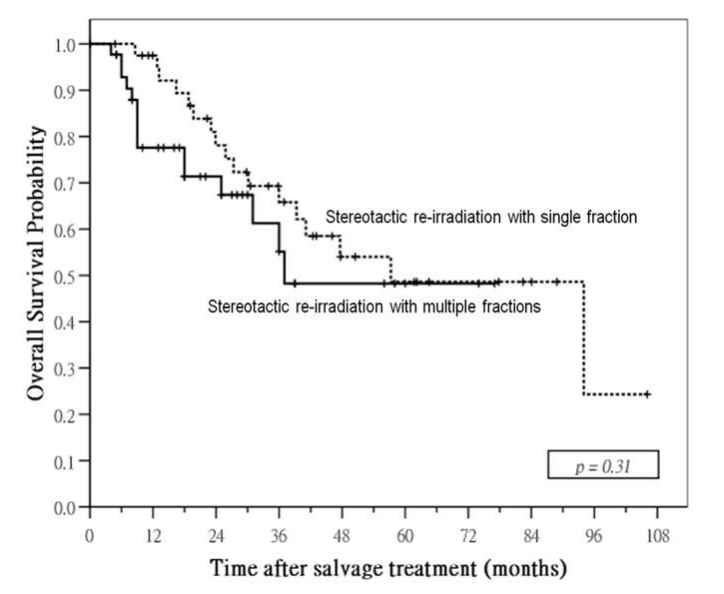
**Survival curves: comparison of overall survival probabilities in patients with local failures of nasopharyngeal carcinoma treated by stereotactic radiotherapy using single or multiple fractions**.

Median follow-up time for surviving patients was 40 months (range: 3 – 106) after SRS and 24 months (range: 5 – 77) after SRM. Local control was significantly better in patients treated by SRM: 1- and 3-year local failure-free rates were 91% and 83% in SRM group compared with 70% and 51% in SRS group (p = 0.003; Figure 3). Nodal relapse was uncommon after salvage treatment in both groups: 3-year nodal-failure free rates in SRM and SRS group were 96% and 85%, respectively (p = 0.19). SRM group had a higher incidence of distant metastases after treatment compared with SRS group, 3-year distant failure-free rate was 69% in the former compared with 82% in the latter, but the difference was not significant (p = 0.089). No significant difference in failure-free rate was observed between the two groups: 1- and 3-year failure-free rates were 75% and 52% in SRM group compared with 62% and 43% in SRS group (p = 0.20). There was also no significant difference in overall survival between the two groups: 1- and 3-year overall survival rates were 78% and 61% in SRM group compared with 98% and 66% in SRS group (p = 0.31; Figure 4).

### Complications

Both SRS and SRM were well tolerated with no severe acute complications. The incidence of severe late complications was higher in SRS group compared with SRM group (33% vs. 21%), although the difference was not statistical significant (p = 0.22). Brain necrosis occurred in 7 patients after SRS (16%) and 5 patients after SRT (12%), with 2 fatal outcome. Massive heamorrhage occurred in 2 patients after SRS (2%) and 1 patient after SRM (4%), with 1 fatal outcome. Altogether there were 3 treatment-related deaths, all occurred in the SRM group.

### Subgroup analysis

**Figure 5:**
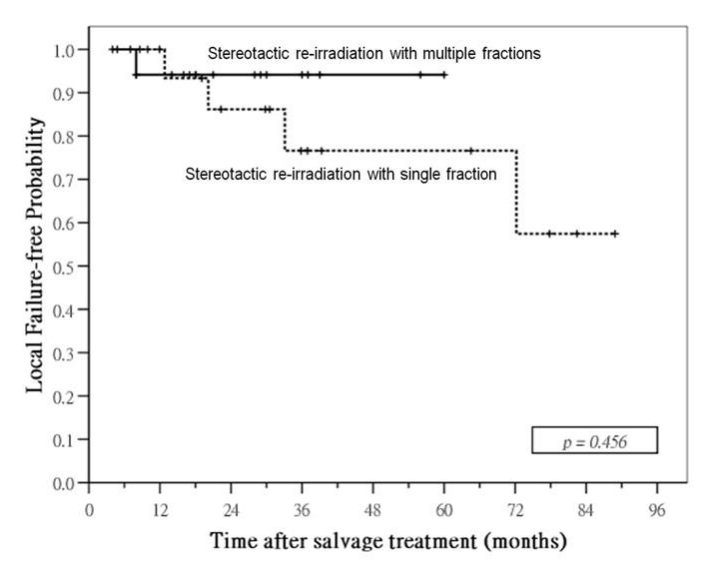
**Persistent disease: comparison of local failure-free probabilities in subgroup of patients with persistent disease**.

**Figure 6:**
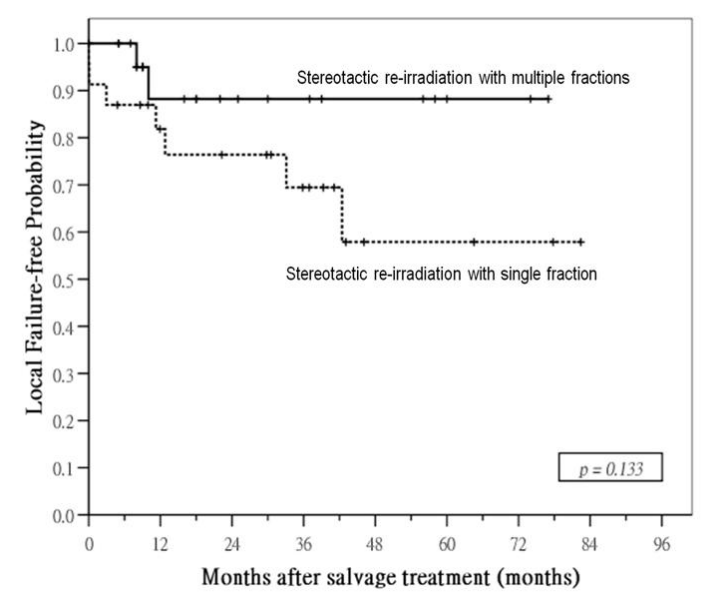
**rT1 tumor: comparison of local failure-free probabilities in subgroup of patients with disease confined to nasopharynx**.

We further analyzed the outcome after SRS and SRM in different subgroups according to important prognostic factors, and the results are summarized in Table 2. The difference in local control between SRS and SRM group was mainly seen in patients treated for recurrent disease and those with disease extended beyond nasopharynx. In patients with persistent disease as well as those with disease confined to nasopharynx, there was no significant difference in local control after SRS or SRM (Figure 5 &6). No differences in survival rates were observed in all subgroups, including those with significant differences in local control rates favoring SRT group.

**Table 2:** Subgroup analysis of treatment outcome after stereotactic radiosurgery or radiotherapy for local failures of nasopharyngeal carcinoma

	3-year local failure-free rate			3-year overall survival rate		
	SRS	SRM	p value	SRS	SRM	p value
Sex						
male	54%	87%	0.006	63%	63%	0.806
female	42%	73%	0.275	75%	24%	0.067

Age						
≤ 45	27%	87%	0.023	63%	44%	0.338
> 45	49%	80%	0.028	67%	62%	0.702

Type of failure						
persistent disease	77%	94%	0.456	87%	90%	0.589
recurrent disease	30%	75%	0.001	52%	35%	0.419

rT stage						
rT1	69%	88%	0.133	85%	79%	0.721
rT2-4	28%	78%	0.006	45%	28%	0.458

Tumor volume						
≤ 5 cc	62%	90%	0.219	79%	75%	0.502
> 5 – 10 cc	61%	67%	0.342	78%	44%	0.117
> 10 cc	69%	84%	0.106	25%	52%	0.108

SRS: stereotactic radiotherapy using single fracton

SRM: stereotactic radiotherapy using multiple fractions

## Discussion

Aggressive treatment of local failure of NPC is generally recommended since a significant proportion of patients can still be successfully salvaged and long-term survivors are not uncommon with reported 5-year survival rates ranging from 54% after surgery [10] to 60–77% after brachytherapy [11,12]. Although surgery and brachytherapy can produce excellent results, only selected cases of local failure of NPC with disease confined to nasopharynx are amenable to these treatments. Most patients with local failure of NPC require external beam radiotherapy but treatment results after re-irradiation using conventional technique remained poor. The reported five-year survival rates after external reirradiation ranged from 7.6% to 36% with the use of conventional two-dimensional treatment planning and radiotherapy (6–8), and 12.4% in a mixed cohort of patients treated either with conventional two-dimensional or three-dimensional conformal radiotherapy [13]. A high incidence of late complication was commonly observed after external beam reirradiation, majority being neurological damage and soft tissue fibrosis. In a cohort of patients with local failure of NPC and all received re-irradiation by three-dimensional conformal technique, the incidence of severe late complications was still high with 5-year actuarial incidence of 100% for ≥ grade 3 toxicity and 49% for ≥ grade 4 toxicity (9).

The concept of applying SRS in the retreatment of NPC is attractive due to the frequent involvement of intracranium and base of skull in NPC and the general radiosensitivity of the tumor. There were several published reports of retreatment of NPC by SRS and the reported tumor control rates ranged from 53 to 86% [14-20], but most of these were small series with a relatively short follow-up. In a previous report based on patients treated by SRS at Queen Mary Hospital, 5-year local failure-free and overall survival rates were 47.2% and 46.9%, respectively [21]. Neuroendocrine complications occurred in 27% of patients but there were no treatment-related deaths. The results of that report compared favorably with that of gold grain implantation based on outcome of patients treated at the same institution [22].

Based on radiobiology principle, fractionation will provide better therapeutic ratio and improve treatment outcome in retreatment of NPC, and SRM has subsequently been explored as a salvage treatment for NPC. Mitsuhashi et al treated 3 patients with rT1 NPC using SRM at a dose of 50–64 Gy, and all 3 patients achieved complete response and remained free of local disease at 4–61 months [23]. The report by Mitsuhashi et al also included another patient with mucoepidermoid carcinoma of the nasopharynx treated by SRM after previous two courses of external radiotherapy, but the treatment was complicated by rupture of the internal carotid artery resulting in patient death. Using SRM at a dose of 24 Gy in 2 to 4 fractions, Orecchia et al reported a less satisfactory outcome in 13 patients with locally recurrent NPC, with a 3-year survival rate of 31% [24]. Ahn et al treated 12 patients with recurrent NPC by SRM using a median dose of 54 Gy, and reported a 2-year local control rate of 92% [25]. Yau et al compared the outcome of 52 patients with NPC treated by either brachytherapy or SRT for persistent disease, and observed a better tumor control after SRM [26]. Xiao et al reported the outcome of 50 patients with persistent or recurrent nasopharyngeal carcinoma treated by SRM with a dose ranged from 14 to 35 Gy using a fraction dose of 5 to 15 Gy [27]. Of the 31 evaluable patients with persistent disease, 94% had complete response with a one-year disease-free survival rate of 47%. Eighteen patients, most of them had rT3-4 tumor, were treated for recurrent disease. The complete response rate was 56% and 1-year disease-free survival rate was 47%. In Xiao's series, however, 16% of patients treated by SRM developed fatal haemorrhage, probably due to the relatively high cumulative dose delivered. The largest published series was from the primary data set of 90 SRT patients used for the current study [28]. The reported 3-year local control rate was 89% for persistent disease and 19% for recurrent disease. Three-year disease-specific survival rate was 58%. The incidence of severe late complications was 19% and there were 3 treatment-related deaths.

Based on matched-pair data from the two largest reported SRS and SRM series for NPC, we demonstrated superior tumor control with SRM, but survival rates were similar. Possible explanations include different follow-up duration in the two groups, the higher incidence of treatment-related deaths in SRM group, the use of additional salvage treatments, and different failure patterns. In SRM groups, no additional radiotherapy was given after local failure due to the high cumulative dose, whereas in SRS group, additional radiotherapy was given whenever possible after documented treatment failure. Thus the use of second salvage treatment may partly account for comparable survival rates in the two groups. In addition, patients in SRM group had a higher incidence of distant metastases than SRS group probably related to the percentage of higher N stage in the former group, and the survival benefits obtained with improved local tumor control were likely to be offset by the occurrence of distant metastases.

Late complications are common in patients receiving re-irradiation for NPC. In view of the high radiation dose already received by patients during prior radiotherapy and the presence of numerous nearby critical structures, it is unrealistic to expect any new form of re-irradiation to be totally risk-free. Late complications, however, differ significantly in terms of incidence and severity among different techniques of re-irradiation. In general, patients with bulky disease and tumor extended beyond nasopharynx usually have a higher incidence of late complications. When patients with similar tumor extent and size are being considered, SRS or SRM usually leads to lower incidence of late complications compared with other techniques because of high dose conformity to the target. One severe and highly fatal complication that can occur after re-irradiation is massive hemorrhage in the nasopharynx, sometimes leading to fatal outcome. The reported incidence of severe hemorrhage after SRS or SRM was relatively high compared to other re-irradiation techniques. Possible causes of severe hemorrhage after re-irradiation include mucosal necrosis, tumor progression, and carotid aneurysm. The latter one is an important cause of uncontrolled bleeding which should not be overlooked. In order to reduce the risk of hemorrhage as a result of carotid aneurysm/rupture following re-irradiation, careful selection of patients and treatment planning are important. Patients with direct tumor encasement of cavernous sinus and internal carotid artery should not be treated by SRS, and the dose to carotid artery should be minimized in all cases. Dose per fraction is also important and most hemorrhage occurred after SRS or SRM using large fractional dose. In patients with tumor encasement of carotid artery, SRM instead of SRS should be used for reirradiation, and a small fractional dose not exceeding 6 Gy is recommended.

The superior tumor control rate achieved by SRM is likely due to the higher dose that can be delivered using this technique compared with SRS. Several reirradiation series have also recognized the important relationship between reirradiation dose and treatment outcome, although the optimal dose is not yet defined. Wang observed reirradiation dose ≥ 60 Gy was associated with improved survival, although most patients received high dose radiotherapy in his series had rT1-2 stage [29]. Similarly, Öksüz et al also reported improved local control and survival after reirradiation with a dose of 60 Gy than < 60 Gy [30]. Lee *et al *also reported improved survival when a reirradiation dose > 60 Gy was used [31]. Teo et al however reported poor survival and high incidence of complications after high dose (≥ 60 Gy) reirradiation of NPC with radical intent, although the survival was still better than those treated with palliative intent using a lower dose of 40–50 Gy [32]. In all these series, retreatment was primarily carried out using conventional two-dimensional radiotherapy. In a cohort of 186 NPC patients reirradiated with either conventional or conformal radiotherapy, Chang et al observed that reirradiation dose ≥ 50 Gy yielded better survival [13]. Using intensity-modulated radiotherapy, Lu et al [33] reported excellent local control rate after high dose (68–70 Gy) retreatment of NPC, although the follow-up time in that study was still short for evaluation of late complications. In another series of reirradiation of NPC also using intensity-modulated radiotherapy, a dose range between 50–60 Gy yielded good tumor control for rT1-3 NPC but not for rT4 disease [34]. Based on these reports, a dose of at least 50 Gy should be delivered using SRM for local failure of NPC, although the optimal fractionation schedule is still not clear. In patients with persistent disease, especially those with small volume disease confined to nasopharynx, a lower dose may be used judging from the results of SRS.

## Conclusion

In conclusion, our study showed that SRM was superior to SRS in salvaging local failures of NPC, especially in patients with recurrent disease and tumor extended beyond nasopharynx. In patients with local failure of NPC, stereotactic re-irradiation using multiple fractions rather than single fraction to deliver a higher total is preferred.

## Competing interests

The authors declare that they have no competing interests.

## Authors' contributions

DC reviewed the treatment records, carried out statistical analysis and drafted the manuscript. SW reviewed the treatment records and assisted in manuscript preparation. VL participated in the design of the study and assisted in the statistical analysis. All authors read and approved the final manuscript. JT assisted in data analysis and drafting the manuscript.

## References

[B1] Chua DT, Sham JS, Wei WI, Ho WK, Au GK (2001). The predictive value of the 1997 American Joint Committee on Cancer Stage Classification in determining failure patterns in nasopharyngeal carcinoma. Cancer.

[B2] Cheng SH, Yen KL, Jian JM, Tsai SY, Chu NM, Leu SY, Chan KY, Tan TD, Cheng JC, Hsieh CY, Huang AT (2001). Examining prognostic factors and patterns of failure in nasophryngeal carcinoma following concomitant radiotherapy and chemotherapy: impact on future clinical trials. Int J Radiat Oncol Biol Phys.

[B3] Au JS, Law CK, Foo W, Lau WH (2003). In-depth evaluation of the AJCC/UICC 1997 staging system of nasopharyngeal carcinoma: prognostic homogeneity and proposed refinements. Int J Radiat Oncol Biol Phys.

[B4] Lee AW, Sze WM, Au JS, Leung SF, Leung TW, Chua DT, Zee BC, Law SC, Teo PM, Tung SY, Kwong DL, Lau WH (2005). Treatment results for nasopharyngeal carcinoma in the modern era: the Hong Kong experience. Int J Radiat Oncol Biol Phys.

[B5] Leung TW, Tung SY, Sze WK, Wong FC, Yuen KK, Lui CM, Lo SH, Ng TY, O SK (2005). Treatment results of 1070 patients with nasopharyngeal carcinoma: an analysis of survival and failure patterns. Head Neck.

[B6] Yan J-H, Hu Y-H, Gu X-Z (1983). Radiation therapy of recurrent nasopharyngeal carcinoma: report on 219 patients. Acta Radiol Oncol.

[B7] Lee AW, Law SC, Foo W, Poon YF, Cheung FK, Chan DK, Tung SY, Thaw M, Ho JH (1993). Retrospective analysis of patients with nasopharyngeal carcinoma treated during 1976–1985: survival after local recurrence. Int J Radiat Oncol Biol Phys.

[B8] Chua DT, Sham JS, Kwong DL, Wei WI, Au GK, Choy D (1998). Locally recurrent nasopharyngeal carcinoma: treatment results for patients with computed tomography assessment. Int J Radiat Oncol Biol Phys.

[B9] Zheng XK, Ma J, Chen LH, Xia YF, Shi YS (2005). Dosimetric and clinical results of three-dimensional conformal radiotherapy for locally recurrent nasopharyngeal carcinoma. Radiother Oncol.

[B10] Wei WI (2000). Salvage surgery for recurrent primary nasopharyngeal carcinoma. Crit Rev Oncol Hemat.

[B11] Choy D, Sham JST, Wei WI, Ho CM, Wu PM (1993). Transpalatal insertion of radioactive gold grain for the treatment of persistent and recurrent nasopharyngeal carcinoma. Int J Radiat Oncol Biol Phys.

[B12] Leung TW, Tung SY, Sze WK, Sze WM, Wong VY, O SK (2000). Salvage brachytherapy for patients with locally persistent nasopharyngeal carcinoma. Int J Radiat Oncol Biol Phys.

[B13] Chang JT, See LC, Liao CT, Ng SH, Wang CH, Chen IH, Tsang NM, Tseng CK, Tang SG, Hong JH (2000). Locally recurrent nasopharyngeal carcinoma. Radiother Oncol.

[B14] Firlik KS, Kondziolka D, Lunsford LD, Janecka IP, Flickinger JC (1996). Radiosurgery for recurrent cranial base cancer arising from the head and neck. Head Neck.

[B15] Miller RC, Foote RL, Coffey RJ, Gorman DA, Earle JD, Schomberg PJ, Kline RW (1997). The role of stereotactic radiosurgery in the treatment of malignant skull base tumors. Int J Radiat Oncol Biol Phys.

[B16] Buatti JM, Friedman WA, Bova FJ, Mendenhall WM (1995). Linac radiosurgery for locally recurrent nasopharyngeal carcinoma: rationale and technique. Head Neck.

[B17] Kocher M, Voges J, Staar S, Treuer H, Sturm V, Mueller RP (1998). Linear accelerator radiosurgery for recurrent malignant tumors of the skull base. Am J Clin Oncol.

[B18] Cmelak AJ, Cox RS, Adler JR, Fee WE, Goffinet DR (1997). Radiosurgery for skull base malignancies and nasopharyngeal carcinoma. Int J Radiat Oncol Biol Phys.

[B19] Chen HJ, Leung SW, Su CY (2001). Linear accelerator based radiosurgery as a salvage treatment for skull base and intracranial invasion of recurrent nasopharyngeal carcinoma. Am J Clin Oncol.

[B20] Pai PC, Chuang CC, Wei KC, Tsang NM, Tseng CK, Chang CN (2002). Stereotactic radiosurgery for locally recurrent nasopharyngeal carcinoma. Head Neck.

[B21] Chua DT, Sham JS, Hung KN, Leung LH, Au GK (2006). Predictive factors of tumor control and survival after radiosurgery for local failures of nasopharyngeal carcinoma. Int J Radiat Oncol Biol Phys.

[B22] Chua DT, Wei WI, Sham JS, Hung KN, Au GK (2007). Stereotactic radiosurgery versus gold grain implantation in salvaging local failures of nasopharyngeal carcinoma. Int J Radiat Oncol Biol Phys.

[B23] Mitsuhashi N, Sakurai H, Katano S, Kurosaki H, Hasegawa M, Akimoto T, Nozaki M, Hayakawa K, Niibe H (1999). Stereotactic radiotherapy for locally recurrent nasopharyngeal carcinoma. Laryngoscope.

[B24] Orecchia R, Redda MGR, Regona R, Nassisi D, Jereczek-Fossa B, Zurrida S, Bussi M, Succo G, Sannazzari G (1999). Results of hypofractionated stereotactic re-irradiation on 13 locally recurrent nasopharyngeal carcinoma. Radiother Oncol.

[B25] Ahn YC, Lee KC, Kim DY, Huh SJ, Yeo IH, Lim DH, Kim MK, Shin KH, Park S, Chang SH (2000). Fractionated stereotactic radiation therapy for extracranial head and neck tumors. Int J Radiat Oncol Biol Phys.

[B26] Yau TK, Sze WM, Lee AW, Yeung MW, Leung KC, Hung WM, Chan WI (2004). Effectiveness of brachytherapy and fractionated stereotactic radiotherapy boost for persistent nasopharyngeal carcinoma. Head Neck.

[B27] Xiao JP, Xu GZ, Miao YJ (2001). Fractionated stereotactic radiosurgery for 50 patients with recurrent or residual nasopharyngeal carcinoma. Int J Radiat Oncol Biol Phys.

[B28] Wu SX, Chua DT, Deng ML, Zhao C, Li FY, Sham JS, Wang HY, Bao Y, Gao YH, Zeng ZF (2007). Outcome of fractionated stereotactic radiotherapy for 90 patients with locally persistent and recurrent nasopharyngeal carcinoma. Int J Radiat Oncol Biol Phys.

[B29] Wang CC (1987). Re-irradiation of recurrent nasopharyngeal carcinoma. Treatment techniques and results. Int J Radiat Oncol Biol Phys.

[B30] Öksüz DÇ, Meral G, Uzel Ö, Çağatay P, Turkan S (2004). Reirradiation for locally recurrent nasopharyngeal carcinoma: treatment results and prognostic factors. Int J Radiat Oncol Biol Phys.

[B31] Lee AW, Foo W, Law SC, Poon YF, Sze WM, O SK, Tung SY, Lau WH (1997). Reirradiation for recurrent nasopharyngeal carcinoma: factors affecting the therapeutic ratio and ways for improvement. Int J Radiat Oncol Biol Phys.

[B32] Teo PM, Kwan WH, Chan AT, Lee WY, King WW, Mok CO (1998). How successful is high dose (≥ 60 Gy) reirradiation using mainly external beams in salvaging local failures of nasopharyngeal carcinoma?. Int J Radiat Oncol Biol Phys.

[B33] Lu TX, Mai WY, The BS, Zhao C, Han F, Huang Y, Deng XW, Lu LX, Huang SM, Zeng ZF, Lin CG, Lu HH, Chiu JK, Carpenter LS, Grant WH, Woo SY, Cui NJ, Butler EB (2004). Initial experience using intensity-modulated radiotherapy for recurrent nasopharyngeal carcinoma. Int J Radiat Oncol Biol Phys.

[B34] Chua DT, Sham JS, Leung LH, Au GK (2005). Reirradiation of nasopharyngeal carcinoma with intensity-modulated radiotherapy. Radiother Oncol.

